# Prospective multi-centre Voxel Based Morphometry study employing scanner specific segmentations: Procedure development using CaliBrain structural MRI data

**DOI:** 10.1186/1471-2342-9-8

**Published:** 2009-05-15

**Authors:** T William J Moorhead, Viktoria-Eleni Gountouna, Dominic E Job, Andrew M McIntosh, Liana Romaniuk, G Katherine S Lymer, Heather C Whalley, Gordon D Waiter, David Brennan, Trevor S Ahearn, Jonathan Cavanagh, Barrie Condon, J Douglas Steele, Joanna M Wardlaw, Stephen M Lawrie

**Affiliations:** 1 The Division of Psychiatry, Centre for Clinical Brain Sciences (CCBS), School of Molecular and Clinical Medicine, University of Edinburgh, Edinburgh, UK; 2 Aberdeen Biomedical Imaging Centre, Division of Applied Medicine University of Aberdeen, Aberdeen, UK; 3 The Department of Clinical Physics and Bioengineering, NHS Greater Glasgow South University Hospitals Division, Glasgow, UK; 4 SFC Brain Imaging Research Centre, SINAPSE Collaboration http://www.sinapse.ac.uk, Division of Clinical Neurosciences, University of Edinburgh, Western General Hospital, Edinburgh, UK; 5 Sackler Institute of Psychological Research, Faculty of Medicine, University of Glasgow, Glasgow, UK; 6 Centre for Neuroscience, Division of Medical Sciences, University of Dundee, Dundee, UK

## Abstract

**Background:**

Structural Magnetic Resonance Imaging (sMRI) of the brain is employed in the assessment of a wide range of neuropsychiatric disorders. In order to improve statistical power in such studies it is desirable to pool scanning resources from multiple centres. The CaliBrain project was designed to provide for an assessment of scanner differences at three centres in Scotland, and to assess the practicality of pooling scans from multiple-centres.

**Methods:**

We scanned healthy subjects twice on each of the 3 scanners in the CaliBrain project with T_1_-weighted sequences. The tissue classifier supplied within the Statistical Parametric Mapping (SPM5) application was used to map the grey and white tissue for each scan. We were thus able to assess within scanner variability and between scanner differences. We have sought to correct for between scanner differences by adjusting the probability mappings of tissue occupancy (tissue priors) used in SPM5 for tissue classification. The adjustment procedure resulted in separate sets of tissue priors being developed for each scanner and we refer to these as scanner specific priors.

**Results:**

Voxel Based Morphometry (VBM) analyses and metric tests indicated that the use of scanner specific priors reduced tissue classification differences between scanners. However, the metric results also demonstrated that the between scanner differences were not reduced to the level of within scanner variability, the ideal for scanner harmonisation.

**Conclusion:**

Our results indicate the development of scanner specific priors for SPM can assist in pooling of scan resources from different research centres. This can facilitate improvements in the statistical power of quantitative brain imaging studies.

## Background

Structural Magnetic Resonance Imaging (sMRI) of the brain is employed in the assessment of a wide range of neuropsychiatric disorders. Voxel Based Morphometry (VBM) has been established as a leading method for analysing large sMRI studies. VBM is a fully automated process that is used to localise differences in brain parenchyma [1,2]. The VBM implementation segments T_1_-weighted MRI scans into voxel-wise mappings of grey and white tissue and Cerebrospinal Fluid (CSF). It provides for statistical comparisons of these mappings within clinical studies. VBM requires good quality co-registration at the voxel level and it is sensitive to differences between MRI scanners. Reports of VBM analyses that pool scans from different sites for analysis have employed validity assessments [3,4]. In a validity assessment, a VBM contrast of control subjects between the contributing sites is used to map the regions of significant difference between scanners. A masking image that charts these regions is formed. These masked regions are excluded from VBM reporting as results in these regions could be driven by artifactual scanner differences [5,6].

In VBM the use of validity masking is undesirable because it limits the analyses to less than whole brain coverage. As VBM draws its inferences from voxel-wise comparisons it is necessary to apply fine grain corrections of the sMRI tissue classification in order to avoid validity masking. We investigated making such corrections by scanning the same fourteen healthy subjects, twice, at three scanning sites in Scotland with T_1_-weighted sequences. These acquisitions were implemented as part of the CaliBrain study, and we have complete sets of scans for thirteen subjects. The three scanners in the CaliBrain project were matched on the basis of vendor, field strength and head coil type. In this investigation we used an established sMRI analysis tool Statistical Parametric Mapping (SPM5) [1] to segment the T1 scans into grey and white tissue maps and CSF maps.

Baseline analyses of the Calibrain T_1 _segmentations revealed significant differences between the scanners and these differences were of an order that would require validity masking. We investigated the practicality of compensating for scanner differences through the adjustments in the SPM5 segmentation procedure. The SPM segmentation protocol employs spatial priors that map the probable distributions of grey and white tissue and CSF. These priors account for the low frequency variability in tissue presentation across the brain. We adjusted these prior mappings to compensate for the scanner differences. In this process we developed separate sets of priors for each scanner. As above, we refer to these as scanner specific priors. In VBM analyses of the segmentations based upon scanner specific priors, we found that the baseline differences which had indicated a requirement for validity masking were removed.

In addition to VBM analyses we applied metric tests to quantify within scanner variability and between scanner differences. These metrics were applied at baseline and on the adjusted segmentations. The metric results demonstrated that the use scanner specific priors can reduce the tissue classification differences between scanners. However, these reductions were not sufficient to bring the between scanner differences down to the level of within scanner variability.

## Methods

### Study Design

The CaliBrain project was designed to allow for the assessment of differences between scanners and for these differences to be considered in the context of within scanner variability. For this, healthy subjects travelled twice to each of the three scanning centres within a six months period. At each visit the subjects received a T_1_-weighted structural MRI scan. We used SPM5 [1] to segment the structural scans into baseline grey and white tissue maps and CSF maps. The SPM priors used in these baseline tissue classifications were taken from a study of psychosis which employed a scan sequence that was equivalent to that used for the CaliBrain acquisitions [7]. The priors in the psychosis study were drawn from scans of young adults with a family history of schizophrenia and control subjects with no family history of psychosis. All 93 subjects in this study were well at time of scanning.

The practice of adjusting the SPM tissue priors specifically for a cohort acquired at one scanning centre is well established [8-12] and the importance of matching the spatial priors to the investigated population was demonstrated in a study of healthy young adults [13]. We have extended the practice of adjusting the SPM priors adjustment so that they provide compensation for scanner differences. To achieve scanner level compensation we implemented an iterative adjustment protocol that employed proportional feedback to develop scanner specific priors for each scanner. We illustrate the operation of this protocol by randomly selecting six subjects from the CaliBrain project. We used the 1^st ^round scans of these subjects to develop the scanner specific priors. These priors were then used to segment all the CaliBrain T_1 _scans and this gave the segmentations for our adjusted analyses. VBM contrast analyses and metrics were used to assess the scanner differences at baseline and for adjusted segmentations. The seven subjects that were excluded from the scanner specific process formed a test group upon which we could assess the viability of our protocol.

### Data Acquisition

The CaliBrain project acquired MRI brain scans from three imaging research centres: The Department of Radiology, University of Aberdeen; The Division of Psychiatry and The SFC Brain Imaging Research Centre within The Centre for Clinical Brain Sciences (CCBS) at The University of Edinburgh; and The Department of Clinical Physics, NHS Greater Glasgow South University Hospitals Division. The three scanners used were manufactured by General Electric (GE Healthcare, Milwaukee, Wisconsin) and had primary field strengths of 1.5T. The scanners are nominated within this report as scanners 'A, B and C'.

Fourteen healthy participants (10 male, mean age 36.3, age range 22–51 years) took part in the study. All participants were native English speakers, right-handed (self reported), met the standard MRI safety criteria and had no history of diagnosed neurological disorder, major psychiatric disorder or treatment with psychotropic medication, including treatment for substance misuse. The participants were not paid, but they were reimbursed for expenses. All participants provided written informed consent and the study was approved by the local research ethics committees. The scan records were incomplete for one subject and thus the harmonisation methods in the CaliBrain project were conducted on the basis of 13 healthy participants.

Three General Electric 1.5T scanners were used in this study, with some inevitable differences in hardware and software versions. In site A scanning was conducted with a General Electrics (GE) 1.5T Signa NVi/CVi scanner (software version 9.1; gradients with max. amplitude 40 mT/m and max. slew rate 150 T/m/s; standard quadrature head coil). In site B scanning was conducted with a General Electrics (GE) 1.5T Signa LX scanner (software version 9.1M4; Echo-speed gradients with max. amplitude 22 mT/m and max. slew rate 120 T/m/s; standard quadrature head coil). In site C scanning was conducted with a General Electrics (GE) 1.5T Signa scanner (software version 11M3/11M4SP1; gradients with max. amplitude 40 mT/m and max. slew rate 150 T/m/s; standard quadrature head coil).

All subjects participated in six scanning sessions, two at each of the three sites. The time lapse between scans at each site was nominally two weeks. The scanning parameters were kept constant across the three scanners, allowing for minor deviations arising from differences in scanner hardware and software. A high resolution T_1_-weighted scan was acquired using a 3D inversion recovery-prepared fast gradient echo volume sequence with the following parameters: orientation coronal; repetition time (TR) of 5.9 ms (sites A and C) or 8.2 ms (site B); echo time (TE) of 1.9 ms (site A) or 3.3 ms (site B) or 1.4 ms (site C); slice thickness = 1.7 mm without a gap; inversion time (TI) 600 ms; matrix = 256 × 256, voxel within slice dimension = 0.86 mm square; field of view (FOV) = 220 mm^2^; flip angle = 15°; 128 slices.

### VBM Preprocessing and Segmentation

Prior to SPM5 segmentation, co-registration and reslicing procedures were applied to ensure that all the scans were aligned to the anterior-posterior commissure axis (AC-PC) in the standard MNI template space. As part of this process the scans were re-sampled to a resolution of 1 × 1 × 1 mm. The SPM5 segmentation at baseline was implemented using a study specific priors set that had been previously developed for a study of psychosis [7]. The SPM5 adjusted segmentations were obtained using the scanner specific priors derived in our priors adjustment procedure. The SPM5 segmentations were run using the default settings, the 'Number of Gaussians per class' was set to [2 2 2] and the 'Bias regularization' was set to 'medium'. In keeping with the established practice in psychosis research the segmented results were output as unmodulated and normalized to the MNI template. The normalization employed the SPM5 default normalization with the 'Nonlinear Frequency Cutoff = 25'. Also, in keeping with established VBM practice for psychosis in tissue density analyses the SPM5 segmentations were smoothed using an isotropic 12 mm Full Width Half Maximum (FWHM) kernel.

### Procedure for creating Scanner Specific Priors

**Figure 1:**
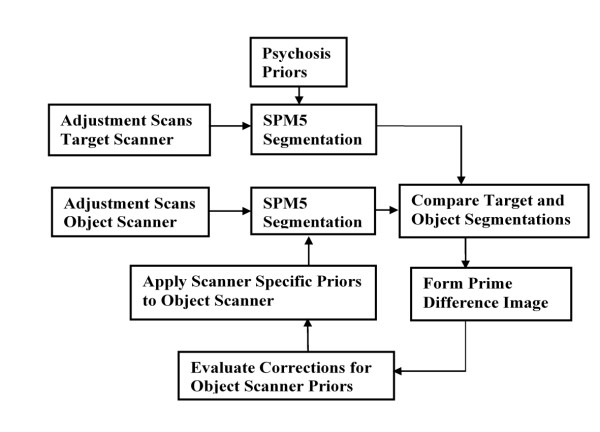
**Process Flow diagram**. Process Flow for procedure that develops scanner specific priors to correct for segmentation differences between the object and target scanners. Adjustment of the object scanner priors is used to minimise the difference between the scanners. The final adjusted object scanner priors are output as the scanner specific priors.

Our iterative procedure that compensates for scanner differences employs proportional feedback to develop sets of scanner specific priors for use in the adjusted SPM5 segmentations. The process flow diagram in Figure 1 gives an overview of this procedure. We designate one scanner as the target scanner and a second as the object scanner. The scans from the target scanner are segmented using SPM5 and for this segmentation the priors were taken from our psychosis study [7]. The priors applied to the target scanner remain unchanged throughout the run of the iterative procedure. The object scanner segmentation is also initialised with the priors taken from our psychosis study [7]. Then through our iterative procedure these object scanner priors are incrementally adjusted. These adjustments are set to compensate for the segmentation differences between the target and object scanners.

The process illustrated in Figure 1 adjusts the object priors through comparisons based upon grey and white segmentations. This dual dependence of the adjusted priors on grey and white tissue is accommodated by allowing the iteration process to alternate the prime comparison between the grey and white tissue types. Thus for every other iteration the prime tissue comparison is applied to the grey segment and this is interspersed with the prime tissue comparison being made on the white segment.

When grey is the prime comparison segment we adjust the grey prior to correct for the voxel level differences found between the target and object scanners and also at the voxel level we apply a balancing adjustment to the white or CSF prior to ensure that the sum of the priors at the voxel level is maintained at its nominal sum of unity. Similarly when the prime comparison is made upon the white segment we adjust the white prior to correct for the differences between the target and object scanner and we apply a balancing adjustment to the grey or CSF prior. In VBM assessments of psychosis CSF presentation is not an established measure of interest, thus we do not assign CSF the prime status within the priors adjustment process.

On a subject by subject basis the prime comparison segmentations obtained from the target and object scanners are subtracted. These subtractions were implemented at the voxel-level and the differences were averaged across the subjects included in the priors adjustment process. The averaged voxel-level differences were used to form a difference image that was then smoothed to suppress sampling noise and reduce subject bias. Next a proportion of the smoothed difference image is used to adjust the grey, white and CSF priors applied to the object scanner. These adjusted priors are then made available for the next iteration of the procedure. This process is repeated until the segmentations given by the object scanner converge with those given by the target scanner. We assess this convergence through the use of metrics described below.

The evaluation of the prime difference image *Pgdiff *for the grey segment *G *is given in equation (1). When the prime comparison segment is white *W*, the difference image *Pwdiff *is given by equation (2). In these calculations of the prime difference images, the processed scans are designated by subscripts *(subject, visit, scanner)*, with *N *= 6, the number of compared subjects. These comparisons were limited to the 1^st ^round scans. The averaging across the adjustment subjects suppresses individual differences.(1)(2)(3)(4)(5)

When the primary segment is grey the adjusted prior *adjGprior *is given by equation (3). In this voxel-level process the current grey prior *curGprior *has a proportion *beta *of the smoothed prime difference *sPgdiff *image subtracted. When the prime comparison segment is grey the evaluations of the adjusted white *adjWprior *and CSF priors *adjCprio*r are given by equations (4) and (5). In these the changes applied to the grey prior are balanced by equivalent additions to the white or CSF priors. At the voxel level we test the relative occupancy of the white and CSF priors and assign the balancing adjustment to which ever prior exhibits greater occupancy. When the prime comparison segment is white the adjusted priors evaluations are equivalent to those given in equations (3), (4) and (5) with the exceptions that the prime difference image is given by *Pwdiff *and the grey and white priors are interchanged. This averaged difference images *Pgdiff *and *Pwdiff *are smoothed using an isotropic kernel, with a FWHM of 10 mm. Smoothing at this level suppresses sampling noise and limits the subject bias that results from the relatively small number of subjects that we have used to create the scanner specific priors

The value of *beta *determines the proportion of the difference image that is used to adjust the priors for the following iteration of the protocol. The setting of *beta *has an important bearing on this protocol. A high setting for *beta *could lead to instability whilst using value that is too low could result in sluggish convergence. As part of the development of this method, we experimented with the *beta *setting and found that setting *beta *to 0.33 or greater could lead to instability in the priors adjustment process. We found that a *beta *setting of 0.15 allowed for stable convergence of the segmentations from different scanners. We found that further reductions of the *beta *value did not improve the degree of convergence that was obtained from the adjustment process. The reductions in *beta *did increase the number iterations required to attain convergence. We ran a between scanner distance metric to assess the degree of convergence between the target and object scanners. We terminated the adjustment procedure when the incremental change in the between scanner distance was less than 0.1% and held at this level in subsequent iterations.

### Testing Scanner Specific Priors Procedure

We tested our priors adjustment procedure by randomly selecting six subjects from the CaliBrain project. We applied the scanner specific priors adjustment to the 1^st ^round scans of these subjects. We designated the scanners in the CaliBrain project as scanners A, B and C. Scanner A was set as the target scanner and scanner B as the object scanner and we developed a set of scanner specific priors for scanner B. We also developed scanner specific priors for scanner C with scanner A set as the target scanner. Throughout these adjustment procedures the priors set used for scanner A was fixed as the priors drawn from our study of psychosis [7]. The scanner specific priors developed for scanners B and C were initialised with the priors from our psychosis study. The choice of scanner A for as the target scanner was based upon the baseline metric results that indicated that scanner A has a low within scanner variability and that it exhibited the lowest overall between scanner differences.

### VBM Statistical Analysis

Using the SPM5 application we implemented VBM statistical analyses of the grey and white segmentations at baseline and for our adjusted segmentations. In these we treated the visits and scanners as separate grouping components and thus formed a factorial analysis matrix that was composed of six groups. We designated the Independence variable as 'NO' to account for the fact that we have repeated measures on the same subjects. We reported the overall F-test for main effect of scanner in the CaliBrain study. Also, we used this design matrix to report t-test contrast results for within scanner variability and between scanner differences. The t-tests for between scanner differences were made by combining the two visits at each scanner. All t-tests and the F-test were carried out with an uncorrected threshold of 0.001, and we reported Family wise error (FWE) correction for multiple comparisons. All groups were composed of the same subjects and as the scans were all acquired within a six month period there was no requirement to covary for age or gender.

### Voxel-wise distance metrics

We employed a percentage distance metric to quantify the within scanner variability and between scanner differences. In SPM, tissue occupancy is assigned at the voxel level for grey, white and CSF as full occupancy or as partial volumes. At full occupancy the voxel is assigned as either grey or white or CSF with occupancy of 1.0. At the interface between tissue types partial occupancy is assigned on a continuous scale from 0.0 to 1.0 and the sum of the assigned occupancy for each voxel does not exceed 1.0.

In order to evaluate the distance between two tissue classifications we computed as a percentage the absolute distance. The general form of the absolute percentage distance computation is illustrated in equation (6) where we compare two voxels V1 and V2. This reports the percentage absolute difference with respect to the average value of the compared voxels. We chose this metric because it accentuates the differences in the compared segmentations.(6)

In keeping with established VBM analyses the metrics were applied to the smoothed segmentations and limited to valid-voxels where the compared segments had occupancy of greater than 0.05. The summary value reported by the metric is an average of the absolute percentage difference found at the valid voxels in the normalised and smoothed segmentations. The metrics are applied on a subject basis and for each subject we evaluate the within scanner variability for scanners A, B and C and we evaluate the between scanner differences for the scanner pairs AB, AC and BC. A paired sample t-test is used to compare the baseline and adjusted metric results and to report the mean difference and its significance.

## Results

### Metric Results

We applied the percentage distance metric to the grey matter segmentations to obtain measures of within scanner variability and between scanner differences. The metric was applied at baseline and after adjustment using the scanner specific priors. Table 1 gives the grey matter metric results averaged across the six subjects used to generate the scanner specific priors. Table 2 gives the grey matter metric results averaged across the seven subjects who were excluded from the process that developed the scanner specific priors. In Tables 1 and 2 we note the mean difference between baseline and adjusted analyses and we give the p_value for the paired sample t-test as measure of significance in the adjustment process.

**Table 1:** Grey matter metric results for the subjects used in priors generation

Scanner Comparison	Baseline*	Adjusted*	Mean Difference (paired sample significance)
AA	2.1 (0.5)	2.1 (0.5)	0.00 (p < 1.00)
BB	3.0 (0.5)	3.0 (0.4)	0.02 (p < 0.79)
CC	2.1 (0.6)	2.1 (0.5)	0.02 (p < 0.36)
			
AB	7.2 (0.8)	3.7 (0.4)	3.5 (p < 0.001)
BC	8.0 (0.8)	4.0 (0.6)	4.0 (p < 0.001)
AC	3.1 (0.4)	2.3 (0.3)	0.8 (p < 0.001)

Grey matter metric results averaged over the six subjects used to generate the scanner specific priors. For the within and between scanner comparison both baseline and adjusted absolute percentage distances are recorded.

*Absolute Percentage distance % (std dev)

**Table 2:** Grey matter metric results for the subjects excluded from priors generation

Scanner Comparison	Baseline*	Adjusted*	Mean Difference (paired sample significance)
AA	2.8 (0.6)	2.8 (0.6)	0.00 (p < 1.00)
BB	3.4 (0.6)	3.4 (0.6)	0.00 (p < 1.00)
CC	2.6 (0.7)	2.5 (0.7)	0.05 (p < 0.078)
			
AB	7.3 (0.6)	5.1 (0.7)	2.2 (p < 0.001)
BC	8.2 (0.8)	5.5 (0.8)	2.7 (p < 0.001)
AC	4.0 (0.6)	3.6 (0.5)	0.36 (p < 0.003)

Grey matter metric results averaged over the seven subjects excluded from the scanner specific priors adjustment procedure. For the within and between scanner comparisons both baseline and adjusted absolute percentage distances are recorded.

*Absolute Percentage distance % (std dev)

### VBM Analyses

We ran VBM baseline and adjusted analyses on the normalised and smoothed segmentations obtained for the seven subjects who were excluded from the prior's adjustment procedure. In these analyses we treat the visits and scanners as separate grouping components and thus form a design matrix composed of six groups. We applied an F-test to consider the main effect of scanner and t-tests to investigate within and between scanner differences. All groups are composed of the same seven subjects and as the scans were all conducted within a six month period there is no requirement to co-vary for age or gender.

### Baseline VBM Results

**Figure 2:**
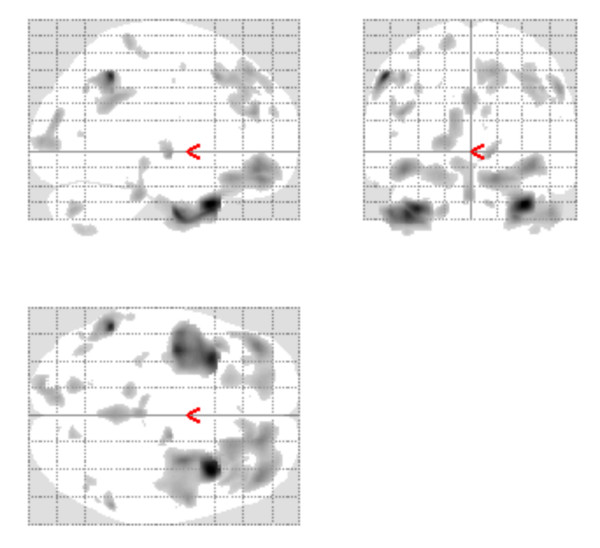
**Grey Matter Baseline Results**. Grey Matter Baseline Maximum Intensity Projection for the CaliBrain Project. Illustrates the regions where the scanners differ when the uncorrected threshold is p < 0.001.

The F-test main effect Maximum Intensity Projection (MIP) for the baseline grey matter analysis is illustrated in Figure 2. The F-test was carried out with an uncorrected threshold of (p < 0.001). This reveals significant scanner effects in the frontal lobes, temporal poles, the thalamus, brain-stem, parietal lobes and occipital lobes. The results of the baseline grey matter F-test are given in Table 3. In this we report the significant maximal voxels giving the MNI coordinate and the anatomical location. We report the Family Wise Error (FWE) corrected p-value of maximal voxel and we also report the extent of the cluster associated with the maximal voxel.

**Table 3:** Grey Matter Baseline maximal voxel results

F-test Cluster Anatomical location	Maximal voxel MNI coordinate	FWE p_corrected
Right Temporal Pole	34, 15, -33	0.001
Left Temporal Pole	-35, 16, -36	0.001
Left Inferior Parietal lobule	-55, -48, 47	0.001
Left Inferior frontal gyrus	-42, 45, -11	0.001
Thalamus	13, -10, -1	0.006
Right Inferior Parietal lobule	56, -48, 40	0.009
Left Middle frontal gyrus	-43, 20, 46	0.014
Left Middle frontal gyrus	-44, 44, 24	0.023
Cingulate gyrus	0, 46, 32	0.040
Brain stem	-1, -30, -28	0.045
Right Superior frontal gyrus	15, 40, 49	0.052

VBM Grey matter baseline tests for the effect of scanner. Reporting the extent of the F-test cluster for an uncorrected threshold of p < 0.001. Giving the anatomical location of the maximal voxel, the MNI coordinate of this maximal voxel and the p_corrected significance.

We investigated the source of these baseline grey matter differences by applying t-test contrasts. t-test comparisons of 1^st ^and 2^nd ^round scans of each scanner revealed that there were no significant within scanner differences. t-test comparisons between the scanners revealed that there were no significant differences between scanners A and C. t-test comparisons between scanners B and C were found to replicate the differences reported in the F-test for main effect of scanner. t-test comparisons between scanners B and A also demonstrated replication of the differences reported in the F-test for main effect of scanner.

**Figure 3:**
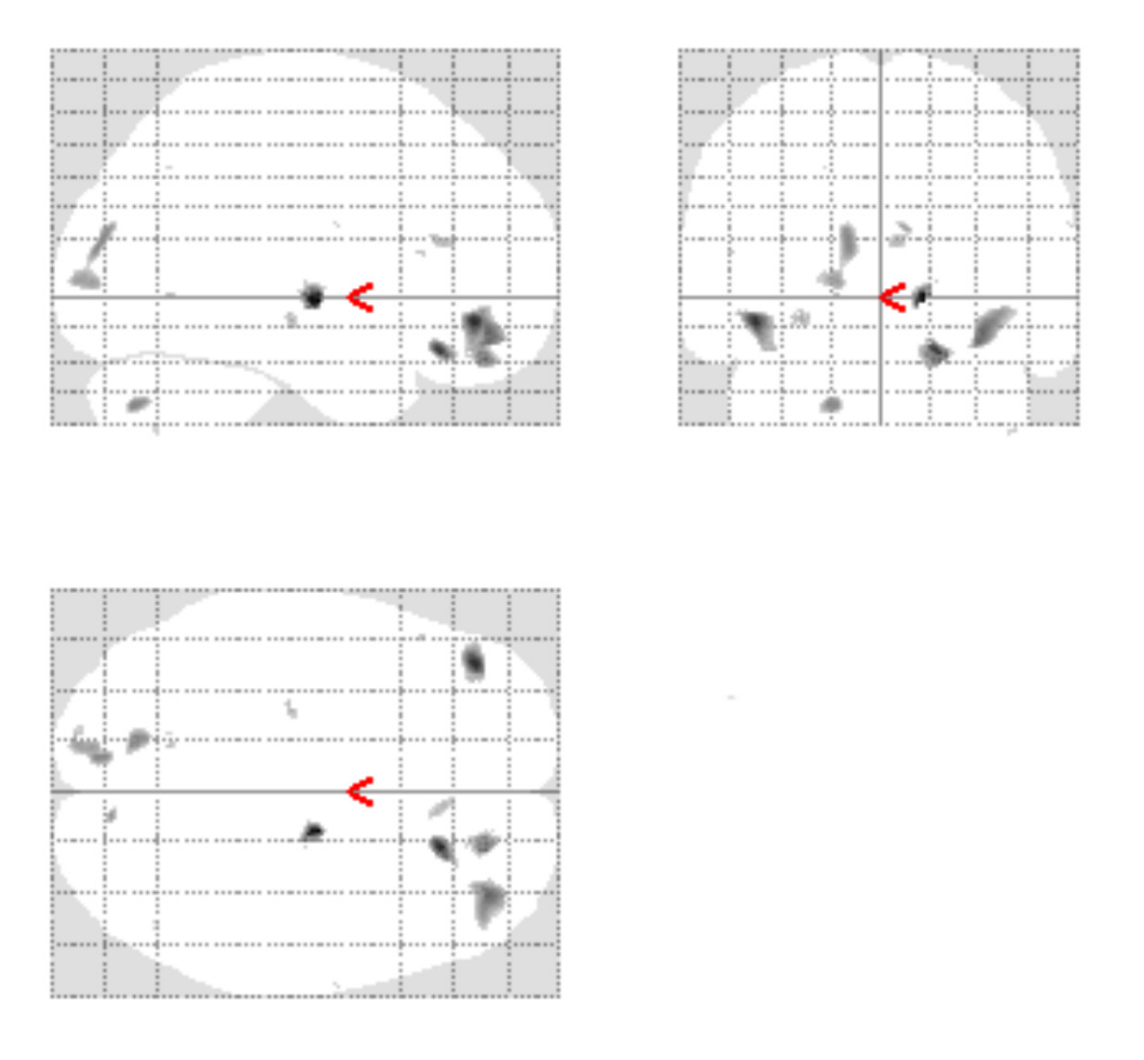
**White Matter baseline Results**. White matter baseline Maximum Intensity Projection for the CaliBrain project, Illustrates the regions where the scanners differ when the uncorrected threshold is p < 0.001.

The F-test results for the baseline white matter VBM analysis are given in Figure 3 and Table 4. This illustrates the spatial distribution of the between scanner differences with significant differences in the right middle frontal gyrus and the thalamus. We investigated sources of these baseline white matter differences by applying t-test contrasts. Comparing 1^st ^and 2^nd ^round scans demonstrated that there were no within scanner differences. In the between scanner tests we found no significant differences for the A-C contrasts and we found significant differences in the A-B and B-C contrasts. The B-C white matter differences were more extensive that those found in the A-B contrasts.

**Table 4:** White Matter Baseline results

F-test Cluster Anatomical location	Maximal voxel MNI coordinate	FWE p_corrected
Right Middle frontal gyrus	18, 45, -19	0.021
Thalamus	15, -10, 0	0.053

VBM White matter baseline tests for the effect of scanner. Reporting the extent of the F-test cluster for an uncorrected threshold of p < 0.001. Giving the anatomical location of the maximal voxel, the MNI coordinate of this maximal voxel and the p_corrected significance.

### Adjusted VBM Results

We applied VBM analyses to the adjusted segmentations obtained from the seven subjects who were excluded from the scanner specific priors development process. The VBM analyses applied were a direct replication of the baseline tests. In these F-tests for the main effect of scanner we found that there were no significant differences for either the grey or white matter analyses. We repeated the adjusted VBM analyses with all 13 CaliBrain subjects for whom we had complete records and these analyses confirmed that no significant differences remained between the pooled scanners.

## Discussion

Combining structural MRI scans from different scanners presents the possibility of increasing the statistical power in VBM analyses of neuropsychiatric disorders. Our aim was to refine the application of SPM segmentation processes and reduce the effects of scanner differences which currently limit multi-centre MRI pooling [3,4]. We have examined the application of the SPM5 priors based segmentation to scans sourced from three scanners. The scanners were matched by vendor, primary field strength, and head coil type, and equivalent sequences were used at each scanner. Although these scanners are well matched we found in our baseline analyses significant between scanner differences in the tissue segmentations. We have demonstrated that if we employ scanner specific priors in our application of SPM that these between scanner differences are reduced.

In VBM analyses the harmonisation constraints for the use of multiple scanners are onerous as VBM requires the provision of corrections for scanner differences at the voxel level. Previous work has shown that it is possible to pool scans from multiple centres in parcellated volumetric studies. In a volumetric analysis of images from multiple scanners [14], their semi-automated method applied global corrections for the tissue classification which were computed separately for each scanner. The methods reported summary volumes for grey and white matter in the cerebrum, and cerebellum and lateral ventricle volumes [15]. This set-level segmentation method employed global estimates of the intensity values that marked the transitions between tissue types and CSF. These globally applied transitions were adjusted for each scanning site.

A methodology that seeks to minimise the differences between scanners through an integration of scan sequence parameters into the segmentation functions was proposed by [16] and gives global adjustment in the intensity to tissue mapping. These global corrections are appropriate in studies where the inferences drawn are limited to lobar tissue occupancy. A volumetric method that addresses the localised intensity to tissue mappings has been proposed by [17] and recognises that localised adjustments for the intensity to tissue mapping within the brain are necessary for scan pooling to be valid for parcellation studies. The method that we have proposed is in keeping with this existing work as we have implemented corrections at a scale that is close to the analysis scale for VBM.

Research for the Alzheimer's Disease Neuroimaging Initiative (ADNI) project demonstrated that to pool scans from multiple sites, it is important to minimise differences between pooled scanners [18-20]. The ADNI project is a longitudinal analysis of ageing and in this within site MRI reproducibility was tested on a range of scanners and sequences. Based upon this research an MR-RAGE sequence was recommended for multiple site scanning and a scheme of corrections that includes field mapping and geometry correction is applied. ADNI researchers investigated the use of B1 field mapping to correct for within scanner variation in the RF inhomogenity for phased array head coils [20]. The results indicated that this technique has limitations. However, B1 field mapping can be applied as an addition to the priors adjustments protocol that we have developed. It is possible that the inclusion of field mapping would further reduce the between scanner differences in the CaliBrain project. However, the scan time acquisitions necessary for correction of the B1 field are not available in the CaliBrain project.

Recent reports of VBM analyses that sourced scans from multiple scanners employed validation masks to limit the reporting of results to regions where the scanner segmentations were equivalent [3,4]. Meda [4] demonstrated in a VBM study of psychosis at four centres that is possible to limit the effects of scanner differences by validity masking and ensuring that in the pooled analysis that the subjects and controls are drawn equally from all contributing centres [4]. A VBM analysis of scans taken from six scanners [3] reported that through the use of equivalent scan sequences and good quality control, the extent of validation masking required could be limited to a single region in the thalamus.

In the CaliBrain project we consider within scanner variability and between scanner differences and our aim was to reduce the between scanner differences to the level of within scanner variability. In keeping with the ADNI recommendations we have sought to minimise the scanner differences in terms of vendor, field strength, head coil and sequences. However, scanner B in the CaliBrain project does differ from scanners A and C in terms of maximum gradient amplitude and maximum slew rate. Our baseline results indicate that scanners A and C are well matched and scans from these two sites could be pooled without further adjustment or compensation. However, our baseline results also demonstrate that scanner B exhibits significant differences with respect to both scanners A and C.

In order to reduce the differences between the scanners in the CaliBrain project we have developed a procedure that employs proportional feedback to adjust the priors for each of the scanners. We have scan records for 13 healthy subjects who were scanned twice at three scanners within a six month period. We demonstrate our protocol for creating scanner specific priors using the 1st round scans of six subjects. We test the adequacy of these scanner specific priors through metric and VBM analyses. The tests for adequacy are applied to the seven subjects who were excluded from the priors adjustment protocol. These tests are limited by the number of subject scans available and we are unable to evaluate the full effects of subject variation expected in a multi-centre clinical study.

Clinical studies that could benefit from the scanner specific priors method are expected to have subject numbers considerably greater than those available for the CaliBrain project. In a multi-centre clinical study, with the exception of the travelling subjects used to develop the scanner specific priors, the subjects would be recruited and scanned independently at the contributing centres. In such a clinical study a test for adequacy of scanner harmonisation could be implemented through comparisons of the healthy control scans recruited from the contributing centres [3,4].

The metric that we report assesses the absolute distance between segmentations. The metrics are applied at the voxel level and are averaged to report an overall distance inclusive of noise and systematic differences. We report in Table 1 on the scans that were used to implement the scanner specific priors procedure. This indicates that the within scanner variability ranges from 3.0% in scanner B to 2.1% in scanners A and C. The adjustment process gives rise to a reduction in the within scanner variability. However, the paired-t tests reveal that these within scanner adjustments do not represent a significant change. In Table 1 the baseline between scanner differences are at a maximum for the BC comparison. Here the adjustment procedure gave rise to significant reductions in all three scanner comparison metrics.

In Table 2 we consider the effects of the scanner specific priors on the scans of seven subjects who were excluded from the priors adjustment process. At baseline the within scanner variability and between scanner distances were equivalent to the baseline results reported in Table 1. Consequently, the use of the scanner specific priors resulted in significant reductions in all three scanner comparisons. However, for the comparisons that include scanner B, the reductions are not sufficient to bring the between scanner difference down to the level of within scanner variability.

The VBM analyses that we applied demonstrated that at baseline there are no significant differences between scanners A and C, However, we found that comparisons of scanners A and C with scanner B gave rise to differences that would require validity mapping such as that employed in VBM analyses by [3,4]. After developing scanner specific priors for scanners B and C and re-segmenting the scans we found that the requirement for validity mapping was removed, because we recorded no significant differences in the grey and white matter F-tests for scanner effect.

## Conclusion

Our results indicate the development of scanner specific priors for the SPM application can assist in the pooling of scan resources from different research centres. This development can facilitate scan pooling and allow for improvements in the statistical power of multi-centre brain imaging studies. Our results indicate that six subjects were adequate for the purpose of matching the scanners in the CaliBrain project. In the typical clinical study the range of tissue presentations would be expected to be greater than that seen in our study of healthy controls. Thus it is likely that in a clinical study that more than six travelling subjects would be required. The number of travelling subjects required would depend upon the diversity of tissue presentation in the study and upon the nature of the differences in the scanners pooled. The method that we have suggested may be limited to multi-site studies in which there are no major hardware and acquisition protocol differences across sites. The CaliBrain project uses scanners from the same vendor all with the same field strengths and head coils with matched sequences. This provides an optimal environment for multiple site scan pooling. Different field strengths and image acquisition protocols could have very different tissue contrasts that would lead to marked differences in segmentation results. In such cases the differences in tissue classification may well be beyond the scope of our compensatory method.

## Competing interests

The authors declare that they have no competing interests.

## Authors' contributions

TWJM developed the scanner correction procedure, played a leading role in the design of CaliBrain study and is the principal author of the manuscript; VEG is the main working researcher on the CaliBrain project, they developed the links with the three contributing scanning centres, established the CaliBrain scanning protocols and contributed to the manuscript; DEJ played a major role in the design of the CaliBrain project, assisted in the development of the scanning protocols at the three centres, and contributed to the final text; AMM assisted in the design of the clinical objects of the CaliBrain project and assisted in the preparation of the manuscript. LR contributed to the statistical analyses and contributed to the final manuscript; GKSL assisted in the development of the multi-centre links, the development of the scanning protocols and contributed to the final manuscript; HCW contributed to the development of the CaliBrain protocols and assisted in the drafting of the manuscript; GDW contributed to the development of the CaliBrain protocols supported the scanning at one of the centres and contributed to the manuscript; DB contributed to the development of the CaliBrain protocols, supported the scanning at one of the centres, and contributed to the manuscript; TSA contributed to the development of the CaliBrain protocols and contributed to the manuscript; JC assisted in the design of the clinical objectives of the CaliBrain project and contributed to the manuscript; BC contributed to the development of the CaliBrain protocols and contributed to the manuscript; JDS assisted in the design of the clinical and methodological objects of the CaliBrain project and contributed to the manuscript; JMW assisted in the design of the clinical and methodological objects of the CaliBrain project and contributed to the manuscript; SML is the principal architect of the CaliBrain Project. All authors contributed to the drafting of the manuscript and all authors approved the final version for publication.

## Pre-publication history

The pre-publication history for this paper can be accessed here:

http://www.biomedcentral.com/1471-2342/9/8/prepub
